# Structural basis for the neutralization of MERS-CoV by a human monoclonal antibody MERS-27

**DOI:** 10.1038/srep13133

**Published:** 2015-08-18

**Authors:** Xiaojuan Yu, Senyan Zhang, Liwei Jiang, Ye Cui, Dongxia Li, Dongli Wang, Nianshuang Wang, Lili Fu, Xuanlin Shi, Ziqiang Li, Linqi Zhang, Xinquan Wang

**Affiliations:** 1 Ministry of Education Key Laboratory of Protein Science, Center for Structural Biology, School of Life Sciences, Collaborative Innovation Center for Biotherapy, Tsinghua University, Beijing, China; 2 Comprehensive AIDS Research Center, Collaborative Innovation Center for Diagnosis and Treatment of Infectious Diseases, School of Medicine, Tsinghua University, Beijing, China; 3 Beijing VDJBio Co., Ltd, Suite B311, 5 Kaituo Road, Zhongguancun BioMedical Garden, Haidian District, Beijing, China; 4 Collaborative Innovation Center for Biotherapy, State Key Laboratory of Biotherapy and Cancer Center, West China Hospital, West China Medical School, Sichuan University, Chengdu, China

## Abstract

The recently reported Middle East respiratory syndrome coronavirus (MERS-CoV) causes severe respiratory illness in humans with an approximately 30% mortality rate. The envelope spike glycoprotein on the surface of MERS-CoV mediates receptor binding, membrane fusion, and viral entry. We previously reported two human monoclonal antibodies that target the receptor binding domain (RBD) of the spike and exhibit strong neutralization activity against live and pesudotyped MERS-CoV infection. Here we determined the crystal structure of MERS-CoV RBD bound to the Fab fragment of MERS-27 antibody at 3.20 Å resolution. The MERS-27 epitope in the RBD overlaps with the binding site of the MERS-CoV receptor DPP4. Further biochemical, viral entry, and neutralization analyses identified two critical residues in the RBD for both MERS-27 recognition and DPP4 binding. One of the residues, Trp535, was found to function as an anchor residue at the binding interface with MERS-27. Upon receptor binding, Trp535 interacts with the N-linked carbohydrate moiety of DPP4. Thus, MERS-27 inhibits MERS-CoV infection by directly blocking both protein-protein and protein-carbohydrate interactions between MERS-CoV RBD and DPP4. These results shed light on the molecular basis of MERS-27 neutralization and will assist in the optimization of MERS-27 as a tool to combat MERS-CoV infection.

A novel coronavirus, now named Middle East respiratory syndrome coronavirus (MERS-CoV), was initially isolated from a patient with acute pneumonia and renal failure in Saudi Arabia in June 2012^1^,^2^. Since then, 1112 laboratory-confirmed cases of MERS-CoV infection, including at least 422 related deaths, have been officially reported to the World Health Organization (WHO) (http://www.who.int/csr/disease/coronavirus_infections/en/). The clinical presentation of MERS-CoV infection ranges from asymptomatic to very severe pneumonia, septic shock, and multi-organ failure, often resulting in death^3^,^4^,^5^,^6^,^7^,^8^,^9^. MERS-CoV belongs to lineage C of the *Betacoronavirus* genus and has a close genetic relationship with bat coronaviruses HKU4 and HKU5^10^,^11^,^12^. The origin and intermediate host of MERS-CoV are still not clear. Gene fragments in bats from Saudi Arabia and Africa have been found to be nearly identical to those of MERS-CoV, suggesting that bats may be the primary reservoir of MERS-CoV^13^,^14^. However, the route of transmission to humans could result from direct or indirect contact with this reservoir or with another intermediate. The prevalence of MERS-CoV-neutralizing antibodies in camels and the recent isolation of MERS-CoV from dromedary camels suggest that camels could be one of the intermediate hosts^15^,^16^,^17^.

Unlike the severe acute respiratory syndrome coronavirus (SARS-CoV) outbreak in 2002–2003, which resulted in ~8000 infections with a case-fatality rate of ~10%^18^, MERS-CoV has limited human-to-human transmission^5^,^8^. Transmission potential analyses have indicated that MERS-CoV has a lower reproduction number (R_0_) than SARS-CoV (0.69 vs 0.80) and has not yet achieved pandemic potential^19^,^20^. However, the sustained epidemic in animal reservoirs would be expected to lead to spill-over into humans^3^. The current case-fatality rate of MERS-CoV infection is estimated to be as high as 30% among hospitalized patients, and no approved pathogen-specific vaccines or antivirals against MERS are available. Therefore, the development of prophylactic and therapeutic reagents is urgently needed to combat MERS-CoV infection.

The envelop spike (S) glycoprotein of coronaviruses, a class I transmembrane protein, is responsible for receptor binding, membrane fusion, and viral entry^21^. The S protein exists as a trimer, with each monomer consisting of a globular membrane-distal S1 domain, a membrane-proximal S2 domain, and a transmembrane domain. The functional cellular receptor for MERS-CoV has been identified as DPP4 (dipeptidyl peptidase 4, also known as CD26)^22^. The receptor binding domain (RBD) is located in the S1 domain of MERS-CoV spike and is responsible for binding to DPP4^23^,^24^. Both our laboratory and another research group recently determined the crystal structure of the RBD in complex with DPP4 and identified RBD residues that are critical for DPP4 binding and viral entry^25^,^26^. Furthermore, neutralizing monoclonal antibodies capable of disrupting the interaction between RBD and DPP4 were identified and characterized^27^,^28^,^29^,^30^. Specifically, we utilized RBD as an antigen bait to screen a non-immune human single-chain variable domain fragment (scFv) library displayed on yeast and identified two neutralizing antibodies MERS-27 and MERS-4^27^. These two antibodies inhibited *in vitro* infection of MERS-CoV live and pseudoviruses with IC_50_ values at nanomolar concentrations^27^, indicating their potential for prophylactic and therapeutic development.

Here we report the crystal structure of MERS-CoV RBD bound to the MERS-27 Fab. The MERS-27 Fab interacts with the receptor binding subdomain of RBD. Its binding epitope on the RBD overlaps with the DPP4 binding site in a small area consisting of four residues^25^. We utilized biochemical, pseudovirus-entry, and neutralization assays to examine the roles of these four residues in MERS-27 recognition and DPP4 binding. We found Trp535 as an anchor residue in the RBD for MERS-27 recognition, and its interaction with N-linked carbohydrates of DPP4 is important for the binding to DPP4 and for viral entry of MERS-CoV.

## Results

### Overall structure of MERS-CoV RBD bound to MERS-27 Fab

**Figure 1:**
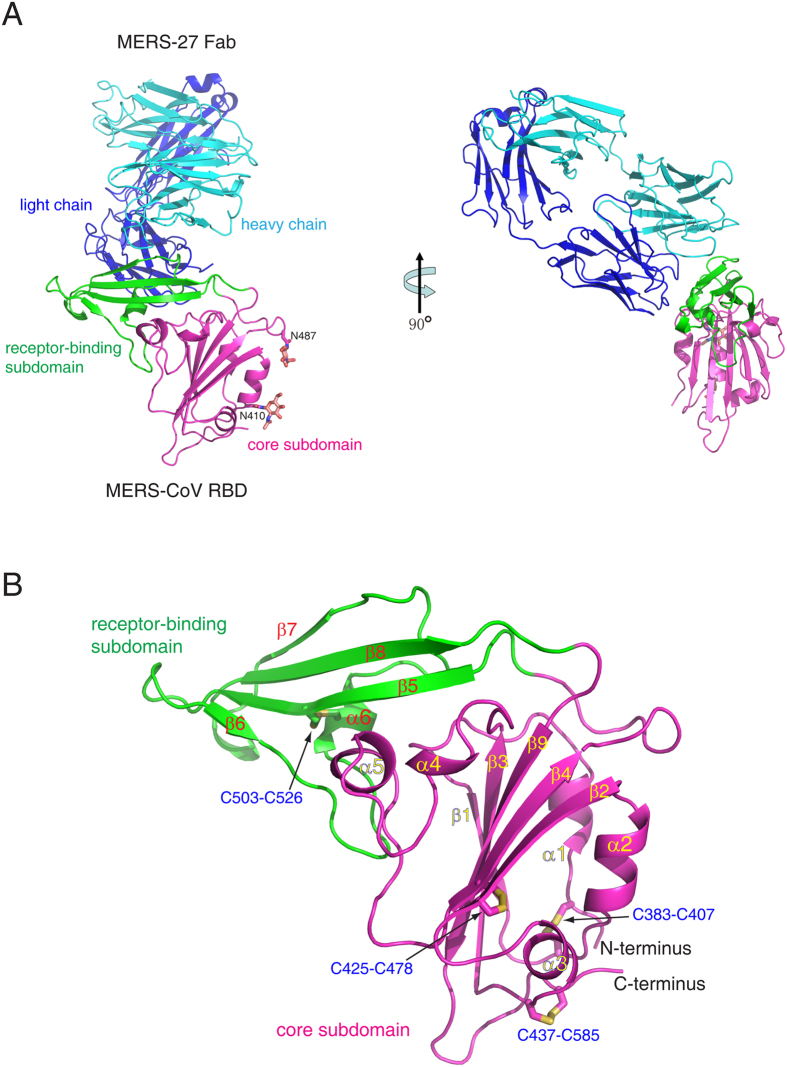
Overall structure. (**A**) Complex of MERS-CoV RBD bound to neutralizing antibody MERS-27 Fab. The RBD core subdomain and receptor-binding subdomain are colored purple and green, respectively. The heavy chain and light chain of MERS-27 are colored cyan and blue, respectively. The N-linked carbohydrates in the RBD are presented as orange sticks. (**B**) Structure of MERS-CoV RBD. The disulfide bonds are presented as yellow sticks.

We generated the RBD of the MERS-CoV spike glycoprotein (residues 367–588) by baculovirus expression in insect cells (Figure S1). This fragment was previously utilized as an antigen bait to isolate the human neutralizing antibodies MERS-4 and MERS-27 from a yeast-displayed single-chain variable region (scFv) fragment library^27^. Recombinant IgG MERS-27 was expressed in HEK293T cells, and the Fab was generated by endoproteinase Lys-c digestion and further purified by size-exclusion chromatography (Figure S1). The MERS-CoV RBD and MERS-27 Fab were mixed *in vitro* to allow the formation of the complex (Figure S1). The complex structure was determined by molecular replacement to a resolution of 3.20 Å (Table 1). In the crystallographic asymmetric unit, the RBD and Fab form two 1:1 binding complexes that are structurally similar with a root-mean-square deviation (RMSD) of 1.17 Å for all 638 Cα pairs (Figure S2). The model consists of residues Val381 to Lys587 of MERS-CoV RBD, Ala1 to Arg210 (light chain) and Val2 to Lys222 (heavy chain) of MERS-27 Fab, and two N-acetyl-D-glucosamine (NAG) molecules attached to Asn410 and Asn487 of RBD, respectively (Fig. 1A).

It has been previously shown that the MERS-CoV RBD comprises the core subdomain and receptor-binding subdomain (Fig. 1A)^25^. Both the heavy and light chain of MERS-27 interacts with the receptor-binding subdomain in the complex (Fig. 1A). The RBD core subdomain comprises a five-stranded anti-parallel sheet (β1, β2, β3, β4, and β9) in the center, with five short helices (α1, α2, α3, α4, and α5) decorating the central β-sheet on both sides (Fig. 1B). The fold is maintained by three disulfide bonds that connect C383 to C407, C425 to C478, and C437 to C585 in the core subdomain (Fig. 1B). The RBD receptor-binding subdomain is a four-stranded anti-parallel sheet (β5, β6, β7, and β8) located between β4 and β9 of the core-subdomain (Fig. 1B). A long loop containing a short helix (α6) connects β6 to β7 in the receptor-binding subdomain (Fig. 1B). The loop crosses the β-sheet perpendicularly on one side, and a disulfide bond between C503 and C526 further links strand β5 to helix α6 (Fig. 1B). The other side of the β-sheet lacks this structural decoration, leaving a relatively flat surface in the receptor-binding subdomain, thereby facilitating interaction with human receptor DPP4. The MERS-CoV RBD structure was previously determined alone and in complex with receptor DPP4^25^,^26^,^31^. The RBD structure was shown to be maintained in the unbound, DPP4-bound, and MERS-27-bound states with three pairwise structural superimpositions showing RMSD for Cα atoms of 0.74, 0.90, and 1.05 Å (Figure S3). Four disulfide bonds maintain the fold in the RBD structure (Fig. 1B). The α1-β1 loop and the α5 helix in the core subdomain can be seen interacting with the β-sheet and the N-terminal segment of the linking loop in the receptor-binding subdomain (Fig. 1B). These features, especially the rigid orientation between the two subdomains, may account for the structural conservation of RBD observed in the different states (Figure S3).

### Binding interface

**Figure 2:**
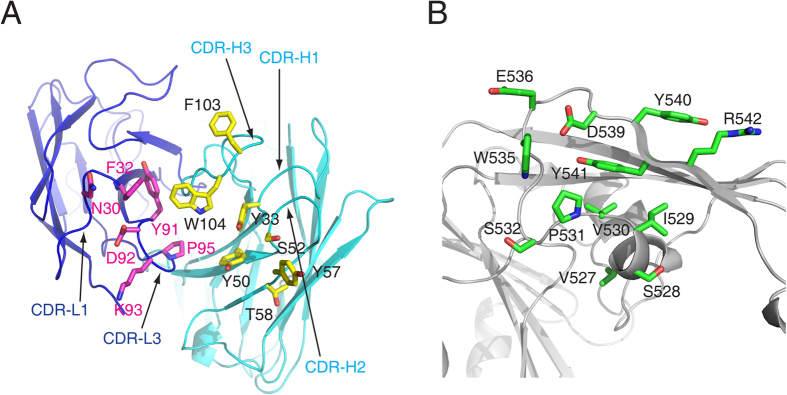
Interacting residues in MERS-27 Fab(**A**) and MERS-CoV RBD (**B**).

The binding interface between MERS-27 Fab and MERS-CoV RBD buries a total of approximately 1384 Å^2^ of surface area, including roughly 408 Å^2^ of the heavy chain, 297 Å^2^ of the light chain, and 679 Å^2^ of the RBD. Residues from the complementary determining regions (CDRs) H1 (Tyr33), H2 (Tyr50, Ser52, Tyr57, and Thr58), and H3 (Phe103 and Trp104) of the heavy chain and L1 (Asn30 and Phe32) and L3 (Tyr91, Asp92, Lys93, and Pro95) of the light chain comprise the interacting residues of MERS-27 (Fig. 2A). The MERS-27 Fab interacts with the C-terminal segment of the linking loop and β7 strand of the receptor-binding subdomain. The epitope on the receptor-binding subdomain consists of Val527, Ser528, Ile529, Val530, Pro531, Ser532, Trp535, Glu536, and Asp539 in the long linking loop and Tyr540, Tyr541, and Arg542 in the β7 strand (Fig. 2B).

**Figure 3:**
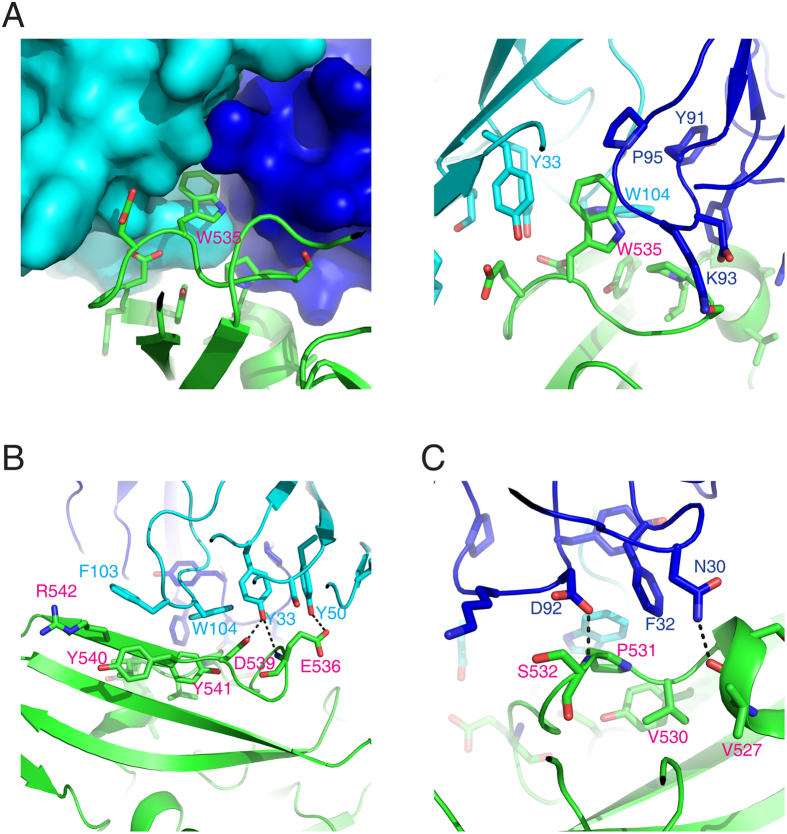
Enlarged focused view of interactions between RBD and MERS-27 Fab. The MERS-CoV RBD is colored green, and the heavy chain and light chain of MERS-27 are colored cyan and blue, respectively.

A cavity is formed by the H1, H2, H3, and L1 CDRs between the MERS-27 heavy and light chains (Fig. 3A). The RBD Trp535 inserts into this cavity and interacts with the surrounding MERS-27 residues Tyr33, Tyr50, and Trp104 of the heavy chain and Tyr91, Lys93, and Pro95 of the light chain (Fig. 3A). Outside the cavity, Phe103 and Trp104 of the heavy chain show hydrophobic interactions with Tyr540, Tyr541, and Arg542 of RBD (Fig. 3B). Heavy chain residues Tyr33 and Tyr50 form hydrogen bonds with Asp539 and Glu536 of RBD, respectively (Fig. 3B). Light chain residue Phe32 shows hydrophobic interactions with Val530 and Pro531 of RBD, while light chain residues Asn30 and Asp92 form hydrogen bonds with main chain atoms of RBD residues Val527 and Ser532, respectively (Fig. 3C).

### MERS-CoV residues critical for both MERS-27 recognition and DPP4 binding

**Figure 4:**
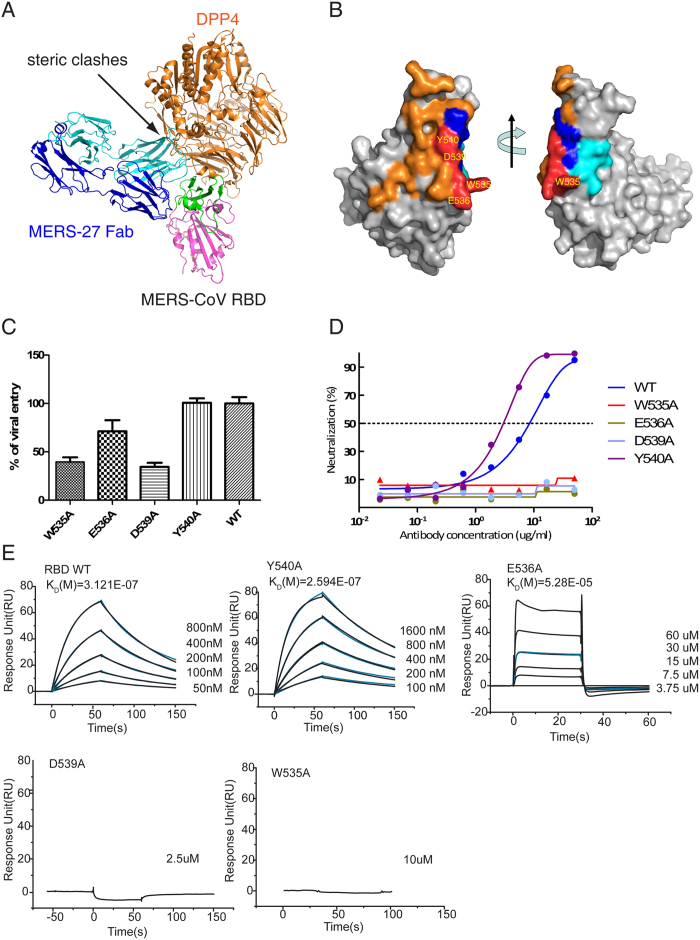
RBD residues critical for MERS-27 recognition and DPP4 binding. (**A**) Structural superimposition of RBD/MERS-27 and RBD/DPP4 complexes. (**B**) Surface representation of DPP4 and MERS-27 binding epitopes in the RBD. The DPP4 binding epitope is colored orange, and the MERS-27 binding epitope is colored blue (heavy chain) and cyan (light chain). The overlapped RBD residues Trp535, Glu536, Asp539, and Tyr540 by these two epitopes are colored red. (**C**) Infection efficiency of MERS-CoV pseudoviruses bearing wild type or mutant spike glycoprotein into Huh7 cells endogenously expressing DPP4. (**D**) Neutralizing activities of MERS-27 against infection of MERS-CoV pseudoviruses bearing wild type or mutant spike glycoprotein into Huh7 cells. (**E**) Binding affinities of wild-type and mutant RBD with MERS-27 Fab.

MERS-27 and DPP4 show steric clashes between the variable domain of the heavy chain and the propeller domain of DPP4 when they simultaneously bound to RBD (Fig. 4A). They bind to the receptor-binding subdomain from different directions (Fig. 4A), resulting in an overlap on an epitope of the receptor-binding subdomain that includes Trp535, Glu536, Asp539, and Tyr540 and that is relatively small compared with the large binding surfaces of MERS-27 and DPP4 on the RBD (Fig. 4B). We previously found that pseudoviruses bearing a triple Glu536Ala/Asp537Ala/Asp539Ala mutant of the MERS-CoV spike glycoprotein had significantly reduced efficiency of viral entry^25^. We performed a more thorough mutagenesis study by introducing single mutations of Trp535, Glu536, Asp539, and Tyr540 into the spike glycoprotein. We first measured the efficiency of entry of pseudoviruses bearing these single-site mutations. We found that Trp535Ala, Asp539Ala, and Glu536Ala mutations reduced the efficiency of viral entry, with the first two mutations (Trp535Ala and Asp539Ala) showing more profound effects (>50% reduction) than the last one (Glu536Ala, ~30% reduction) (Fig. 4C). In contrast, a mutation at Tyr540 had negligible effects on the efficiency of viral entry (Fig. 4C). We then further examined the effects of these mutations on resistance to MERS-27 neutralization. Pseudoviruses bearing spike glycoproteins with Trp535Ala, Glu536Ala, or Asp539Ala mutations failed to be neutralized by the MERS-27 antibody, whereas cell entry by pseudoviruses with the Tyr540Ala mutation was still inhibited by MERS-27 (Fig. 4D). Finally, we examined effects of these RBD mutations on the binding of MERS-27 Fab by using surface plasmon resonance (SPR). Wild type RBD bound the MERS-27 Fab with an affinity of 312 nM (Fig. 4E). The Glu536Ala mutation reduced the binding affinity by 170-fold to 52.8 μM, and the Tyr540Ala mutant did not affect the binding, showing an affinity of 259 nM (Fig. 4E). The individual Trp535Ala and Asp539Ala mutations reduced binding to a level that was undetectable by SPR (Fig. 4E). In sum, results of viral entry, neutralization, and binding assays consistently indicated that Trp535 and Asp539 are critical for both MERS-CoV spike glycoprotein recognition by MERS-27 and binding with receptor DPP4.

### Structural basis for neutralization by MERS-27

**Figure 5:**
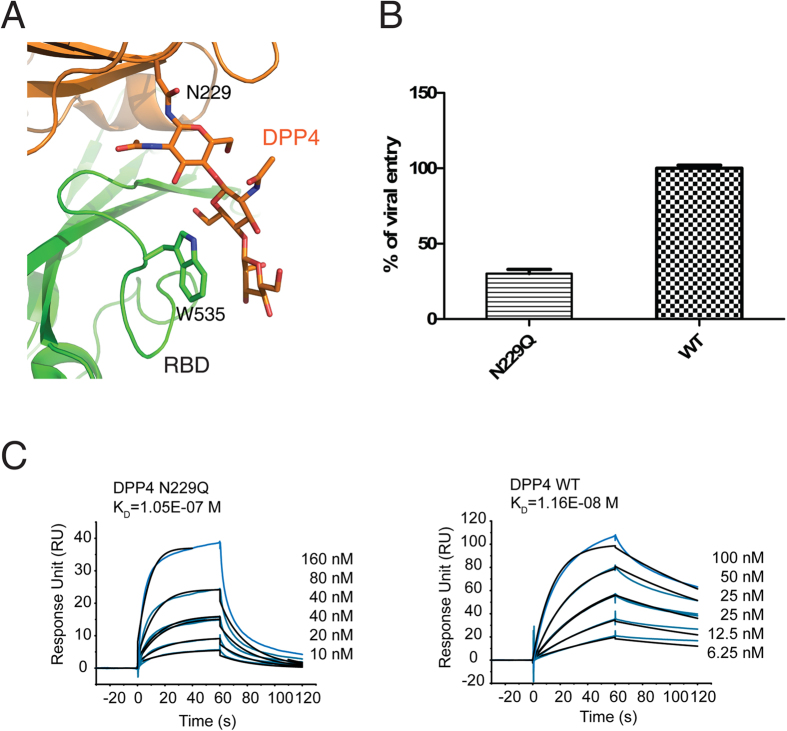
Inhibition of the protein-carbohydrate interaction between RBD and DPP4 by MERS-27 through targeting RBD residueTrp535. (**A**) Closer-up view of the interaction between Trp535 and the DPP4 Asn229-linked carbohydrate moiety^25^,^26^. (**B**) Infection efficiency of MERS-CoV pseudoviruses into COS7 cells expressing wild-type or Asn229Gln mutant DPP4. (**C**) Binding affinities of RBD to wild-type and Asn229Gln mutant DPP4.

In this study, we determined that the two RBD residues Trp535 and Asp539 are critical for both MERS-27 recognition and DPP4 binding (Fig. 4). Asp539 forms a hydrogen bond with heavy chain residue Tyr33 upon MERS-27 recognition and it forms a salt-bridge with Lys267 of DPP4 upon receptor binding^25^ (Figure S4). Therefore, the binding of MERS-27 antibody prevents the formation of the Asp539-Lys267 salt bridge that is important for the receptor binding and viral entry (Fig. 4C). RBD residue Trp535 inserts its side chain into the cavity of MERS-27 (Fig. 3A), forming an anchor at the interface of MERS-27 recognition. In contrast to Asp539 and other RBD residues involved in DPP4 binding, Trp535 does not directly interacts with DPP4 residues. Instead, Trp535 has close contacts with the carbohydrate moiety linked to residue Asn229 of DPP4 (Fig. 5A). Taken together, these results indicate that the protein-carbohydrate interaction might be important for DPP4 binding and the viral entry of MERS-CoV. To further confirm this possibility, we generated a human DPP4 Asn229Gln mutant and measured its ability to bind to RBD and to mediate the cell entry of pseudoviruses. We found that the efficiency of viral entry into COS7 cells expressing the DPP4 Asn229Gln mutant was significantly reduced (~70% reduction) compared with COS7 cells expressing wild-type DPP4 (Fig. 5B). Similarly, the RBD binding affinity was also reduced by approximately 10 fold from the 11.6 nM for wild-type DPP4 to 105 nM for the DPP4 Asn229Gln mutant (Fig. 5C). Therefore, although the MERS-27 binding epitope overlaps with a small area of the DPP4-binding site on RBD, MERS-27 lodges RBD Trp535 in the cavity between its heavy and light chains (Fig. 3A) and forms hydrogen bond with RBD Asp539 (Fig. 3B). In this way, MERS-27 disrupts both the protein-protein and protein-carbohydrate interactions between RBD and DPP4, resulting in the inhibition of MERS-CoV viral entry.

## Discussion

MERS-CoV is the second reported example of a zoonotic coronavirus that results in severe respiratory infection with high mortality rate in humans after SARS-CoV. Therefore, MERS-CoV has attracted significant attention in basic research and clinical studies since its discovery in 2012^1^,^4^. After revealing the structural basis for the specific binding of MERS-CoV spike glycoprotein to the cellular receptor DPP4 and identifying two human neutralizing monoclonal antibodies^25^,^27^, we report here the structural, biochemical, and cellular mechanism of MERS-CoV inhibition by one antibody MERS-27.

Notably, our study revealed that MERS-27 inhibits both protein-protein and protein-carbohydrate interactions between MERS-CoV RBD and DPP4. Although the close contact of Trp535 with the DPP4 Asn229-linked carbohydrate moiety has been previously observed^26^, the role of the interaction in receptor binding and viral entry of MERS-CoV has remained unclear. We showed that the recognition of RBD by MERS-27 is anchored at RBD residue Trp535 through structural and biochemical studies. The interaction between Trp535 of RBD and the DPP4 carbohydrate is also important for receptor binding and viral entry of MERS-CoV. The critical RBD residues considered to be involved in receptor binding and viral entry should include Trp535, which was not defined in our previous study^25^. Notably, recent studies showed that the isolated RBD of the HKU4 spike was able to bind DPP4 and pseudoviruses bearing HKU4 spike infected cells through DPP4^32^,^33^. Structural determination also found that the DPP4 Asn229-linked carbohydrate moiety also interacts with the RBD of HKU4^33^. These results further underscore that the Asn229-linked carbohydrate moiety is important for facilitating the binding of DPP4 to the RBD of MERS-CoV and its close relatives.

In addition to MERS-27, the scFv library screen against MERS-CoV RBD identified another neutralizing antibody, MERS-4. MERS-4 and MERS-27 exhibited a synergistic inhibitory effect on the entry of pseudoviruses^27^. Previously, we generated a series of mutants of the MERS-CoV spike glycoprotein with residues involved in DPP4 binding and analyzed the escape of these mutant-bearing pseudoviruses from antibody neutralization. The MERS-4 epitope largely overlapped with DPP4 binding site^27^. However, none of the mutants that efficiently escaped MERS-4 neutralization showed similar effects with MERS-27, indicating that MERS-4 and MERS-27 recognize different epitopes^27^. The DPP4 binding site in the RBD has been separated into patch 1, which includes Tyr499, Glu536, Asp537, and Asp539, and patch 2, which includes the hydrophobic Leu506, Trp553, Val555 and the surrounding hydrophilic Asp510, Arg511, and Glu513^25^. In this study, we showed that MERS-27 epitope mainly overlaps with the patch 1 DPP4-binding site. Upon MERS-27 binding, more patch 2 DPP4-binding RBD residues are still exposed. Structural data for MERS-4 with RBD have not yet been made available, though we hypothesize that MERS-4 binding targets and blocks the RBD residues in patch 2, which are separated from the MERS-27 epitope in patch 1 and could explain the synergistic effect of MERS-27 and MERS-4^27^.

Other groups also reported the identification of ten human and one mouse neutralizing monoclonal antibodies targeting the RBD of MERS-CoV spike glycoprotein^28^,^29^,^30^. The structural information of these antibodies with RBD has not yet made available. Epitope mapping showed that the mouse antibody Mersmab1 recognizes Leu506, Asp510, Arg511, Glu513, and Trp553 in patch 2^30^. MERS-CoV spike mutations escaped neutralization by seven human neutralizing antibodies, as reported by Tang *et al.*^28^. Nearly all of the mutated residues mapped to patch 2, with the exception of one mutation in the S2 domain of spike^28^. Ying *et al.* reported that Asp510 and Trp553 of patch 2 are important for the binding of RBD to all three of the identified human neutralizing antibodies^29^. The binding epitope of one antibody, m336, includes Glu536 and Asp539, which are also present in the epitope of MERS-27. However, Trp535, which has been shown to be important for MERS-27 binding, was not included in the binding epitope of m336^29^. MERS-27 seems to differ from other identified MERS-CoV neutralizing antibodies in that Trp535 is used as an anchor recognition site, blocking protein-carbohydrate as well as protein-protein interactions.

In summary, our study reported here revealed the structural basis for the neutralization of MERS-CoV by MERS-27. The structural analysis of MERS-27 with MERS-CoV RBD further led us to delineate the importance of the protein-carbohydrate interaction in the binding of RBD to the MERS-CoV cellular receptor DPP4. Our study therefore provides important information required for a complete understanding of MERS-CoV neutralization by different antibodies, which will in turn assist the optimization of these antibodies to ultimately combat MERS-CoV infection.

## Methods

### Protein expression and purification

The MERS-CoV receptor-binding domain (RBD) (residues 367 to 588 of the spike glycoprotein) and the ectodomain of human DPP4 (residues 39 to 766) were expressed in *Spodoptera frugiperda* Sf9 insect cells using the Bac-to-Bac baculovirus expression system (Invitrogen). Sf9 insect cells were maintained in Insect-Xpress protein-free medium (Lonza) without serum at 27 °C. The cDNAs encoding the MERS-CoV RBD and DPP4 ectodomain were cloned into pFastBac-Dual vector with an N-terminal gp67 signal peptide to facilitate secretion and a C-terminal His-tag to facilitate purification. The plasmid was transformed into bacterial DH10Bac competent cells, and the extracted bacmid was transfected into Sf9 cells in the presence of Cellfectin II reagent (Invitrogen). After incubation of the transfected cells at 27 °C for 7 days, the low-titer baculoviruses were harvested by collecting the cell-culture supernatant with centrifugation at 2,000 rpm for 10 minutes. After two rounds of amplification, the high-titer viruses were used to infect Sf9 cells at a density of 2 × 10^6^ cells per milliliter. The cell-culture supernatant containing the MERS-CoV RBD or DPP4 ectodomain were harvested 60 hours after infection, concentrated, and buffer-exchanged to HBS buffer (10 mM Hepes, pH 7.2, and 150 mM NaCl). The MERS-CoV RBD or DPP4 ectodomain was captured with nickel beads, eluted with 500 mM imidazole in HBS buffer, and purified by gel-filtration chromatography using a Superdex 200 High Performance column (GE Healthcare) with the HBS running buffer. The purified MERS-CoV RBD was digested with endoglycosidase F1 and F3 at room temperature overnight and was then further purified by gel-filtration chromatography.

The genes encoding the heavy and light chain of MERS-27 were separately cloned into antibody expression vectors containing the constant regions of IgG1 (kindly provided by Prof. M. C. Nussenzweig of Rockefeller University). The MERS-27 IgG was expressed in HEK293T cells by transient transfection into adherent cells in the presence of polyethylenimine (PEI) (Sigma). The cell-culture supernatant was collected 72 hours after transfection, and the MERS-27 IgG was collected and captured by protein A Sepharose (GE Healthcare). The bound MERS-27 IgG was eluted with 100 mM sodium phosphate buffer (pH 3.0) containing 150 mM NaCl and was then further purified by gel-filtration chromatography using a Superdex 200 High Performance column (GE Healthcare). The purified MERS-27 IgG was digested with endoproteinase Lys-c at 37 °C for 16 hours, and the resulting Fab and Fc fragments were separated by loading the sample onto a DEAE ion-exchange column with 25 mM Tris buffer (pH 8.0). The Fab fragment was collected in the flow through.

### Crystallization and data collection

The purified MERS-CoV RBD and MERS-27 Fab were mixed at a molar ratio of 1:1, incubated for 2 hours at 4 °C, and then purified by gel-filtration chromatography using the Superdex 200 High Performance column (GE Healthcare). The complex was collected and concentrated to ~10 mg/ml in HBS buffer for crystallization. Crystals were successfully grown at 18 °C using the sitting drop vapor diffusion method, which involved mixing equal volumes of protein and reservoir solution containing 0.1 M MES monohydrate (pH 6.5) and 12% (w/v) polyethylene glycol 20,000. All crystals were cryoprotected by soaking in reservoir solution supplemented with 20% glycerol for several seconds and then were cooled in liquid nitrogen. Diffraction data were collected on the BL17U beamline at the Shanghai Synchrotron Research Facility (SSRF) and processed with HKL2000^34^. All data collection and processing statistics are listed in Table 1.

### Structural determination and refinement

The structure was determined by molecular replacement with the crystallographic software PHASER^35^. The search models are the MERS-CoV RBD structure (PDB ID: 4L72) and the structures of the variable and constant domain of the heavy and light chains available in the PDB with the highest sequence identities. Iterative refinement with the program PHENIX and model building with the program COOT were performed to complete the structure refinement^36^,^37^. Structure validation was performed with the program PROCHECK^38^, and all structural figures were generated with PYMOL39^39^. All structure refinement statistics are listed in Table 1.

### Surface plasmon resonance analysis

Real-time binding and analysis by surface plasmon resonance (SPR) were conducted on a BIAcore T200 instrument (GE Healthcare) at 25 °C. The MERS-27 Fab was immobilized on a research-grade CM5 sensor chip by the amine-coupling method. Flow cell 1 was left blank as a reference. MERS-27 Fab (10 μg/mL) in 10 mM sodium acetate pH 5.5 was immobilized to 400 response units on the flow cell 2. For the collection of data, MERS-CoV RBD and its mutants were injected in a buffer of 10 mM Hepes, pH 7.2, 150 mM NaCl, and 0.005% (vol/vol) Tween-20 over the flow cells at various concentrations with a 30 μL/min flow rate. The MERS-CoV RBD and MERS-27 Fab complex was allowed to associate for 60 seconds and dissociate for 90 seconds. Data were analyzed with the BIAcore T200 evaluation software by fitting to a 1:1 Langmuir binding model.

### MERS-CoV pseudovirus production, viral infection, and neutralization assay

The MERS-CoV pseudoviruses were generated in HEK293T cells by co-transfection of the human immunodeficiency virus backbone expressing firefly luciferase (pNL43R-E-luciferase) and the MERS-CoV spike glycoprotein expression vector (pcDNA3.1+, Invitrogen) into HEK293T cells. The mutant spike glycoprotein expression vectors were generated using site-directed mutagenesis, and mutations were confirmed by sequencing. The viral supernatant was harvested 48 hours after transfection and was then normalized using a p24 ELISA kit (Beijing Quantobio Biotechnology Co., LTD, China).

MERS-CoV pseudoviruses bearing wild-type or mutant spike glycoprotein were used to infect Huh7 target cells endogenously expressing hDPP4. The infected Huh7 cells were lysed 48 hours after infection and viral entry efficiency was quantified by comparing the luciferase activities of the pseudoviruses bearing the mutant and the wild-type MERS-CoV spike glycoproteins. For viral infection into COS7 cells, the cells were transiently transfected with pcDNA3.1+ vector encoding wild-type or mutant hDPP4. The transfected COS7 cells were then incubated with goat anti-hDPP4 polyclonal antibody (R&D) followed by incubation with fluorescein phycoerythrin (PE)-labeled rabbit anti-goat IgG antibody (Santa Cruz). The expression levels of wild-type and mutant hDPP4 were measured by flow cytometry (BD Aria II), and the mean fluorescence intensity (MFI) was analyzed. The COS7 cells infected by MERS-CoV pseudoviruses were lysed 4 hours after infection and viral entry efficiency was quantified by comparing the luciferase activities of pseudovirus-infected COS7 cells expressing wide-type and those expressing mutant hDPP4.

Neutralization assays were performed by incubating 100TCID50 (median tissue culture infectious dose) of pseudoviruses with 16 serial 1:3 dilutions of purified antibody at 37 °C for 1 hour. Huh7 cells (about 1.5 × 10^4^ per well) were then added to the antibody and virus mixture. Infectivity was quantified by the luciferase activity 48 hours after infection. IC50s were calculated using the dose-response inhibition model in GraphPad Prism (GraphPad Software Inc.).

## Additional Information

**Accession codes:** The coordinates and structural factors have been deposited into the RCSB Protein Data Bank. The PSB accession code is 4ZS6. 

**How to cite this article**: Yu, X. *et al.* Structural basis for the neutralization of MERS-CoV by a human monoclonal antibody MERS-27. *Sci. Rep.* **5**, 13133; doi: 10.1038/srep13133 (2015).

## Supplementary Material

supplementary figures

## Figures and Tables

**Table 1:** Crystallographic data collection and refinement statistics.

Data collection	
Beamline	SSRF BL17U
Wavelength	0.9796 Å
Space group	*P*2_1_
Cell dimensions	
a, b, c (Å)	81.49, 64.46, 186.05
α, β, γ (°)	90, 100.43, 90
Resolution (Å)	50-3.20 (3.27-3.20)
*R*_merge_ (%)	17.0 (87.6)
I / σI	8.5 (3.0)
Completeness (%)	95.1 (96.7)
Redundancy	5.0 (5.1)
Refinement	
Resolution (Å)	37.0-3.20 (3.27-3.20)
No. Reflections	31129 (2576)
*R*_work_ / *R*_free_ (%)	19.2/24.0
No. atoms	
Protein	9798
Glycan	56
B-factors (Å^2^)	
Protein	73.1
Glycan	79.0
r.m.s. deviations	
Bond lengths (Å)	0.011
Bond angles (°)	1.508
Ramachandran plot (%)	
Most favored	84.1
Additionally allowed	14.8
Generously allowed	0.9
Disallowed	0.2

*R*_work_ and *R*_free_ are defined by *R *= Σ_*hkl*_||*F*_obs_| − |*F*_calc_||/Σ_*hkl*_|*F*_obs_|, where *h*, *k*, and *l* are the indices of the reflections (used in refinement for *R*_work_; 5%, not used in refinement for *R*_free_) and *F*_obs_ and *F*_calc_ are the structure factors, deduced from intensities and calculated from the model, respectively.
