# Supplementary material for: Structural basis for the neutralization of MERS-CoV by a human monoclonal antibody MERS-27
Source: Sci Rep. 2015 Aug 18;5:13133. doi: 10.1038/srep13133 (PMC4539535; doi:10.1038/srep13133)

Structural basis for the neutralization of MERS-CoV by a human monoclonal antibody MERS-27

Xiaojuan Yu[1, *], Senyan Zhang[1, *], Liwei Jiang[2, *], Ye Cui[1], Dongxia Li[3], Dongli Wang[1], Nianshuang Wang[1], Lili Fu[2], Xuanlin Shi[2], Ziqiang Li[3], Linqi Zhang[2, #], Xinquan Wang[1, 4, #]

[1]Ministry of Education Key Laboratory of Protein Science, Center for Structural Biology, School of Life Sciences, Collaborative Innovation Center for Biotherapy, Tsinghua University, Beijing, China

[2]Comprehensive AIDS Research Center, Collaborative Innovation Center for Diagnosis and Treatment of Infectious Diseases, School of Medicine, Tsinghua University, Beijing, China

[3]Beijing VDJBio Co., Ltd, Suite B311, 5 Kaituo Road, Zhongguancun BioMedical Garden, Haidian District, Beijing, China

[4]Collaborative Innovation Center for Biotherapy, State Key Laboratory of Biotherapy and Cancer Center, West China Hospital, West China Medical School, Sichuan University, Chengdu, China

[*]Equal contribution

[#]Corresponding authors

Xinquan Wang: xinquanwang@mail.tsinghua.edu.cn

Linqi Zhang: zhanglinqi@mail.tsinghua.edu.cn

Figure S1. Gel filtration profiles and SDS-PAGE gels of purified MERS-CoV RBD, MERS-27 Fab, and the RBD-Fab complex.

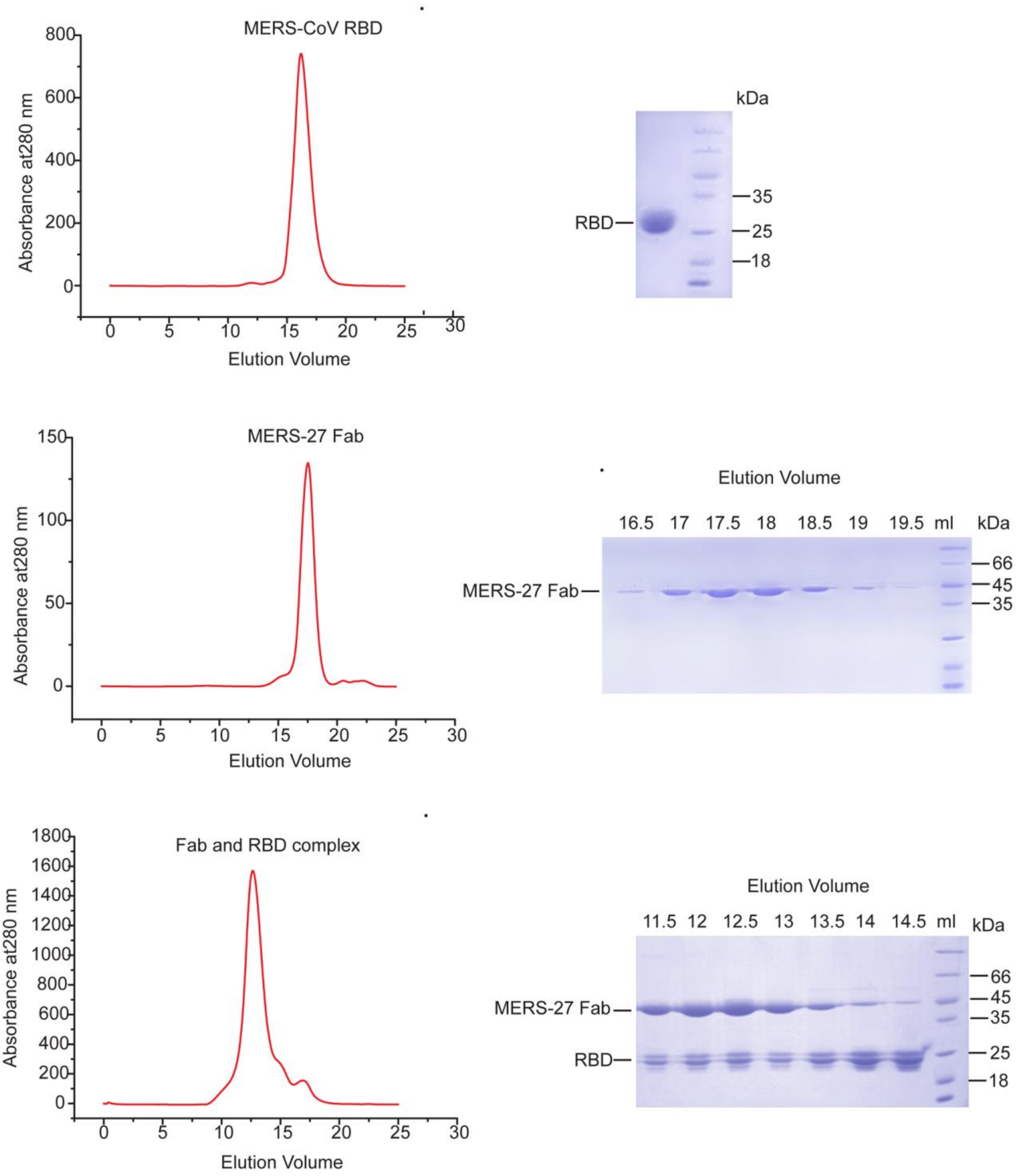

Figure S2. Stereo view of the two complexes of MERS-CoV RBD with MERS-27 Fab in the crystallographic asymmetric unit.

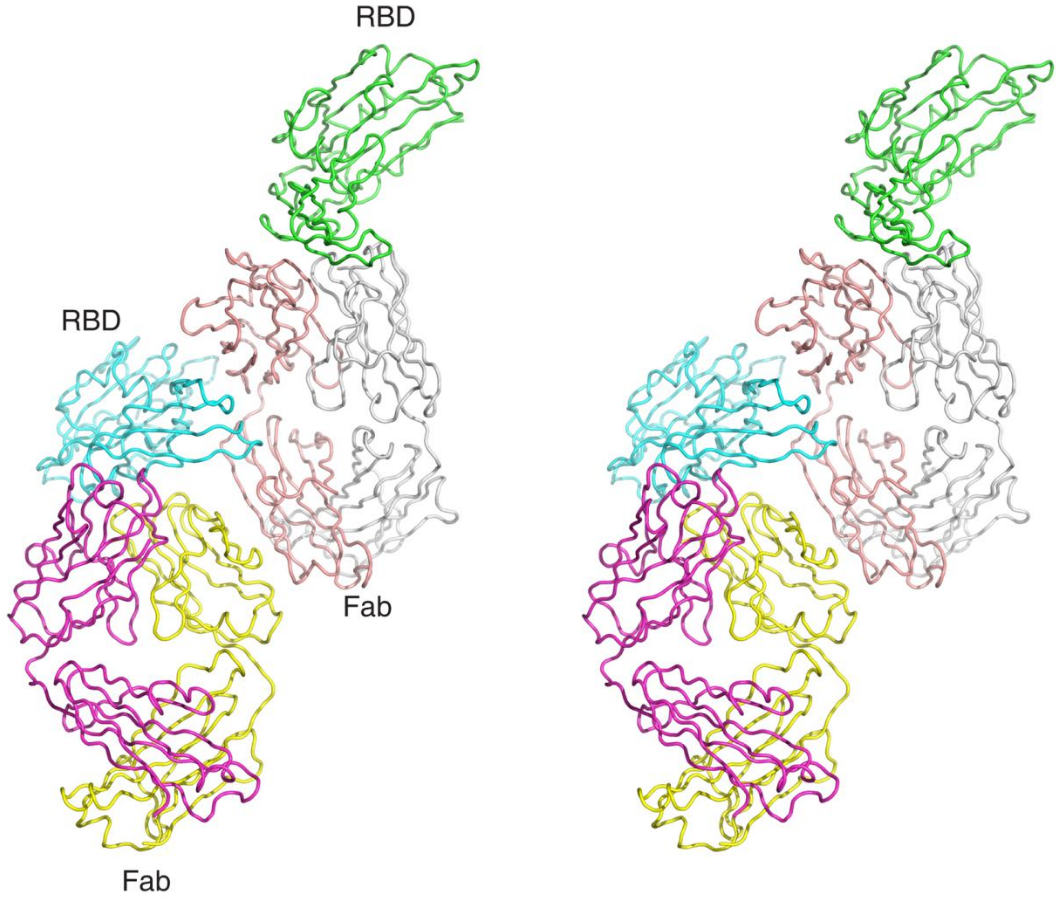

Figure S3. Structural superimposition of RBD in unbound (blue), DPP4-bound (orange), and MERS-27-bound (green) states.

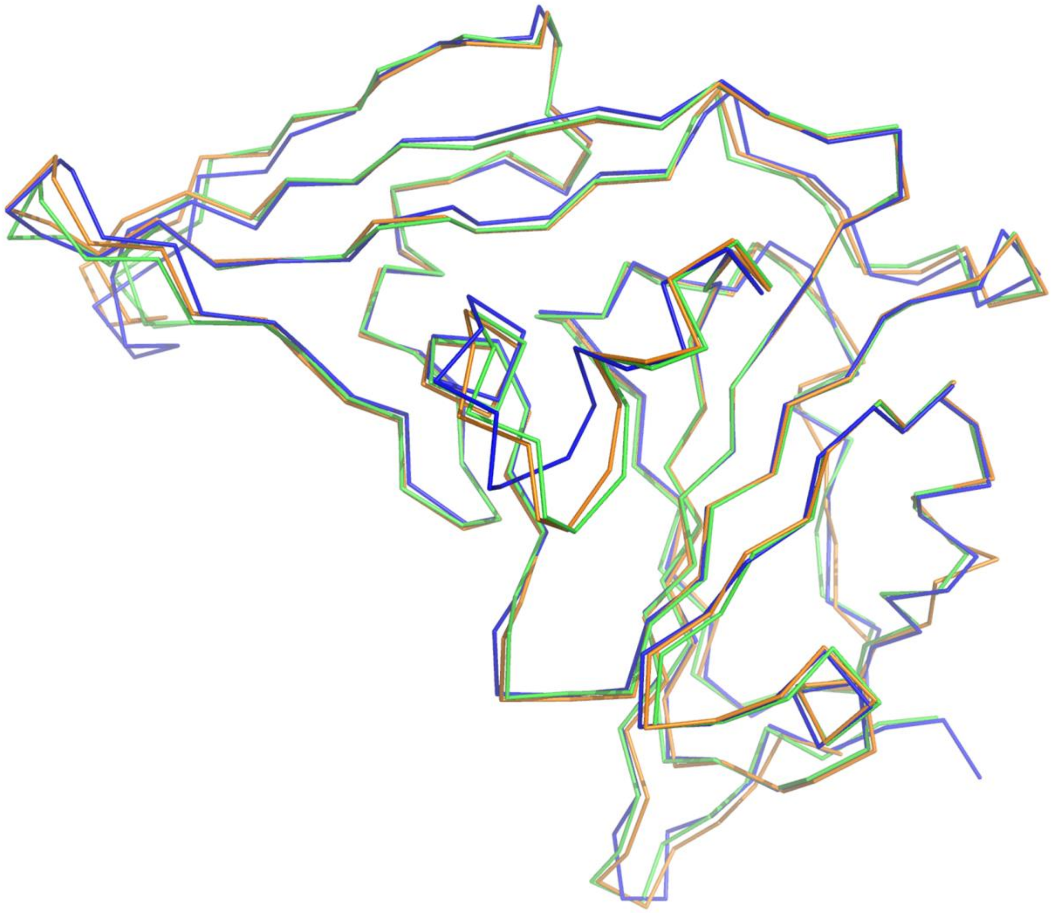

Figure S4. The MERS-CoV RBD residue Asp539 forms a hydrogen bond with Tyr33 of heavy chain upon MERS-27 binding (**A**). This RBD residue forms a salt bridge with Lys267 upon receptor DPP4 binding (**B**).

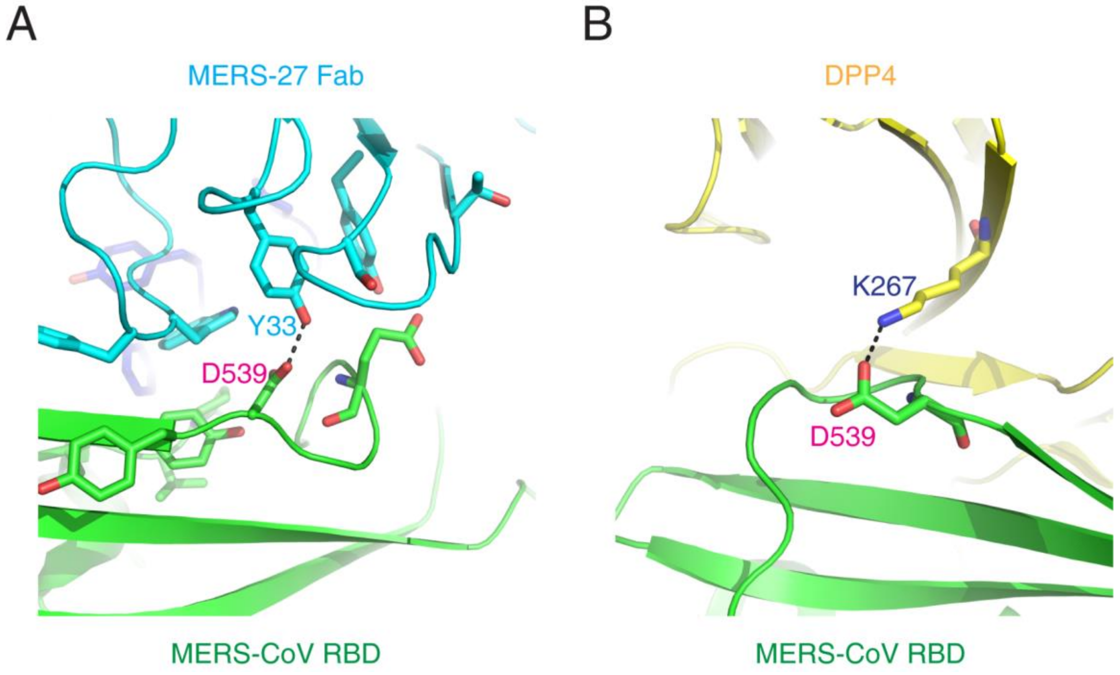

Supplement: supplementary figures [file srep13133-s1.pdf]
